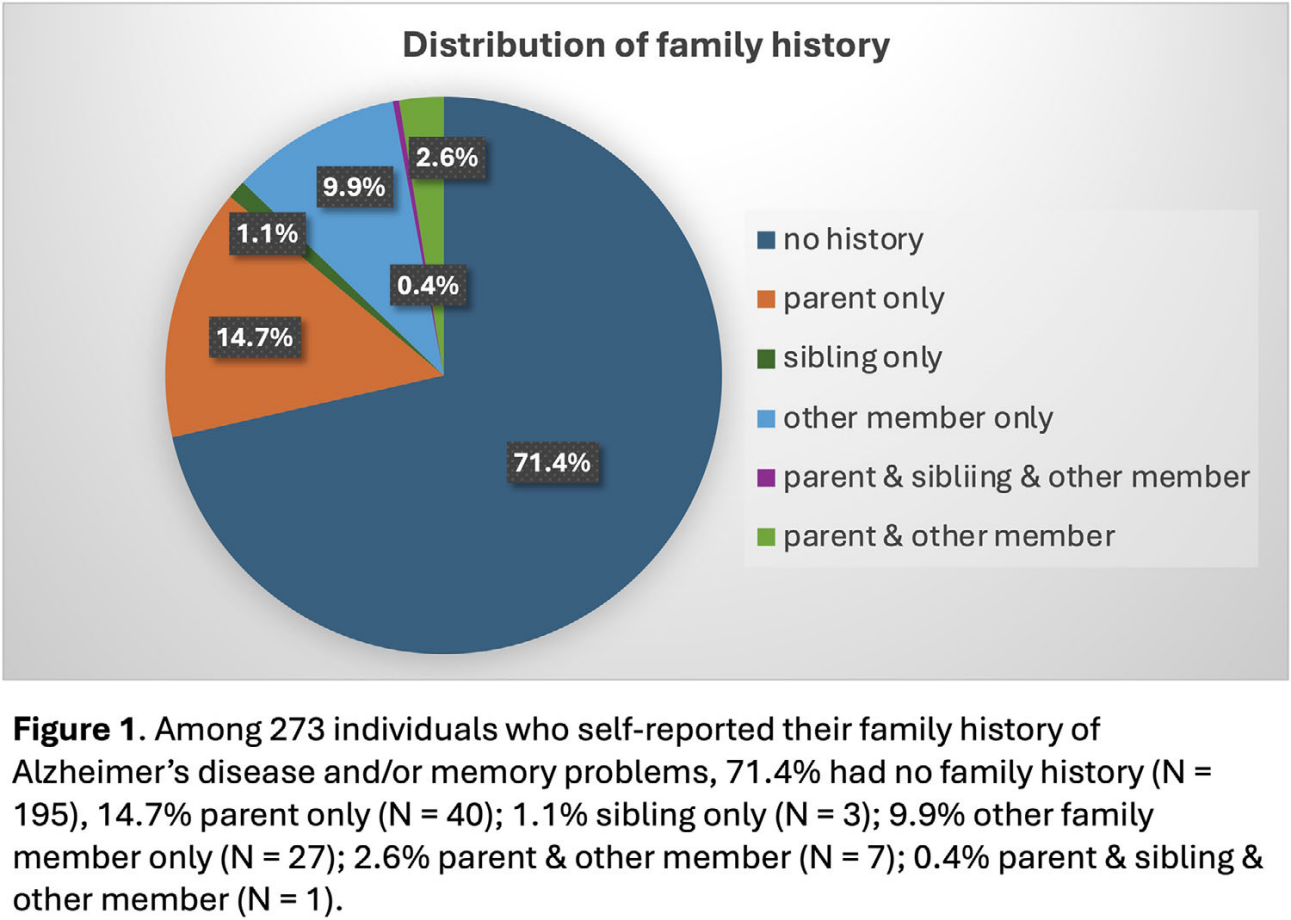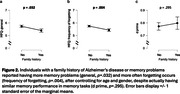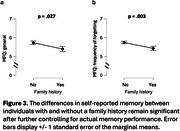# Family history of AD and memory problems associated with worse subjective memory, not objective memory, in cognitively normal individuals

**DOI:** 10.1002/alz.091150

**Published:** 2025-01-03

**Authors:** Mingzhu Hou, William J. Jagust, Xi Chen

**Affiliations:** ^1^ University of Texas at Dallas, Richardson, TX USA; ^2^ University of California, Berkeley, Berkeley, CA USA; ^3^ Stony Brook University, Stony Brook, NY USA

## Abstract

**Background:**

Family history of Alzheimer’s disease (AD) or undiagnosed memory problems is linked to an increased risk of dementia. Subjective memory complaints are also more common among individuals with positive family history, which could be indicative of heightened awareness of memory deficits in these people. Here, we conducted an online study in cognitively normal individuals across the lifespan and aimed to examine whether the presence of family history is linked to worse objective memory performance that mediates the lower subjective memory in these individuals.

**Method:**

Participants’ objective memory was measured online by an object‐scene‐pair memory task that assessed their memory in different domains. Here we focused on the d prime (Z_Hit_‐Z_FA_) for the object‐scene pairs, while other measures yielded similar results. Participants’ subjective memory was assessed by Memory Functioning Questionnaire (MFQ). We focused on the two most commonly used measures from this questionnaire, the general question and the frequency of forgetting subscale, as the primary measures of subjective memory. Participants’ family history was based on self‐report information using a comprehensive family history questionnaire. Family history positivity was defined as any family member having been diagnosed with AD or having undiagnosed memory problems.

**Results:**

Among 273 participants (aged 22‐70 yrs), 71.4% reported no family history (N = 195). In the 78 reports of positive family history, 14.7% was parent only (N = 40), 1.1% was sibling only (N = 3), 9.9% was other family member only (N = 27), 2.6% was parent & another member (N = 7); 0.4% was parent & sibling & another member (N = 1) (Fig.1). Individuals with family history reported having more memory problems (general, p = .032; Fig.2a) and more often forgetting (frequency of forgetting, p = .004; Fig.2b), after controlling for age and gender, despite having equivalent objective memory performance (p = .295; Fig.2c). These associations remained significant while further controlling for actual memory performance in the same model (Fig.3).

**Conclusion:**

Our findings suggest that, contrary to our hypothesis, family history of AD and memory problems is associated with worse subjective memory, but not objective memory, in cognitively normal individuals. Low subjective memory in people with family history may reflect their general worry about dementia that is not based on actual cognition.